# Reduction of TMAO level enhances the stability of carotid atherosclerotic plaque through promoting macrophage M2 polarization and efferocytosis

**DOI:** 10.1042/BSR20204250

**Published:** 2021-06-02

**Authors:** Weihao Shi, Yijun Huang, Zhou Yang, Liang Zhu, Bo Yu

**Affiliations:** 1 Department of Vascular Surgery, Shanghai Huashan Hospital, Fudan University, Shanghai 200040, China; 2 Department of General Surgery, Shanghai Pudong Hospital, Fudan University Pudong Medical Center, 2800 Gongwei Road, Pudong, Shanghai 201399, China

**Keywords:** efferocytosis, methimazole, plaque stability, polarization, trimethylamine N-oxide

## Abstract

It has been demonstrated that trimethylamine N-oxide (TMAO) serves as a driver of atherosclerosis, suggesting that reduction of TMAO level might be a potent method to prevent the progression of atherosclerosis. Herein, we explored the role of TMAO in the stability of carotid atherosclerotic plaques and disclosed the underlying mechanisms. The unstable carotid artery plaque models were established in C57/BL6 mice. L-carnitine (LCA) and methimazole (MMI) administration were applied to increase and reduce TMAO levels. Hematoxylin and eosin (H&E) staining, Sirius red, Perl’s staining, Masson trichrome staining and immunohistochemical staining with CD68 staining were used for histopathology analysis of the carotid artery plaque. M1 and M2 macrophagocyte markers were assessed by RT-PCR to determine the polarization of RAW264.7 cells. MMI administration for 2 weeks significantly decreased the plaque area, increased the thickness of the fibrous cap and reduced the size of the necrotic lipid cores, whereas 5-week of administration of MMI induced intraplate hemorrhage. LCA treatment further deteriorated the carotid atherosclerotic plaque but with no significant difference. In mechanism, we found that TMAO treatment impaired the M2 polarization and efferocytosis of RAW264.7 cells with no obvious effect on the M1 polarization. In conclusion, the present study demonstrated that TMAO reduction enhanced the stability of carotid atherosclerotic plaque through promoting macrophage M2 polarization and efferocytosis.

## Introduction

Atherosclerotic diseases, including acute coronary syndromes and stroke, are the leading causes of mortality and disability in developing countries [1,2]. Noticeably, atherosclerotic carotid artery stenosis consists of 20% of all the etiological factors of ischemic stroke in the globe [3]. In addition to atherosclerotic plaque size, plaque stability is a major risk factor of stroke, which has been raised as a main concentrated issue in clinic targeting carotid stenosis diseases [4,5]. The vulnerable plaques are characterized by a large cholesterol-rich core, low level of calcium and present with thin fibrous caps, with higher risk of stroke [6]. Therefore, it is essential to search potent methods to enhance the stability of carotid atherosclerotic plaque [2].

Atherosclerosis is a complex, multifactorial disease in which multiple cell types are involved, including macrophages [7,8]. During the pathogenesis of atherosclerotic plaques, circulating monocytes move to and reside in the subendothelium of vessel walls and then transform into macrophages, which subsequently transform to foam cells after intaking oxidative low-density lipoprotein (LDL). There are two phenotypes of macrophages in the plaque lesions, one is the proinflammatory phenotype (M1) and other is the anti-inflammatory phenotype (M2). The M1 and M2 phenotypes of macrophages can transform for each other under the stimulation of many factors, such as lipoproteins, lipids, cytokines, chemokines and small bioactive molecules [9]. Evidence has shown that M1 macrophages are mainly derived from the inflammatory Ly6C^high^ monocyte subset, while the M2 macrophages are chiefly derived from Ly6C^low^ monocyte subsets [10]. LPS and interferon-γ (IFNγ) promote M1 activation, leading to increased expression of proinflammatory factors, such as TNF-a (tumor necrosis factor-a), iNOS (nitric oxide synthase 2) and IL-6 (interleukin-6), which are crucial for plaque rupture [11]. To the contrary, IL-4 and IL-13 promote the activation of M2, which contributes to tissue repair and endocytic clearance through secreting anti-inflammatory factors, including arginase-1 (Arg1), mannose receptor C type-1 (Mrc1), interleukin-10 (IL-10), macrophage scavenger receptor 1 (MR), chil3 (YM1) and (Pparg) [12]. In addition, efferocytosis, which refers to the capacity of macrophages to clean up the dead cells, also plays a crucial role in lesion stage and plaque stability [13,14].

Recently, increasing studies have shown that plasma trimethylamine N-oxide (TMAO) level was elevated in atherosclerosis [15–18]. Further evidence identified that TMAO accelerated atherosclerosis [19–22], suggesting that reduction of TMAO level might be a potent method to prevent the progression of atherosclerosis. For example, Ding et al. [19] reported that administration of TMAO in apoE^−/−^ mice induced a 2-fold increase in the total plaque area, together with increased levels of triglyceride (TG), total cholesterol (T-CHO) and low-density lipoprotein cholesterol (LDL-C). However, TMAO role in the stability of carotid atherosclerotic plaque and the underlying mechanisms still remain to be elucidated.

In the present study, we explored the effects of L-carnitine (LCA), a quaternary ammonium compound which can produce TMAO, and methimazole (MMI), an inhibitor of flavin monooxygenase 3 (FMO3) which triggers TMAO formation on the stability of carotid atherosclerotic plaque *in vivo*. Additionally, we explored TMAO effects on the polarization and efferocytosis of RAW264.7 macrophage.

## Materials and methods

### Experimental animals

All animal experiments in the present study were approved by the ethic committee of Huashan hospital, Fudan University (No. 2020-Huashan hospital-JS-322). Sixty-six male ApoE^−/−^ mice at C57/BL6 background (6–8 weeks) were purchased from the Institute of Animal Models of Nanjing University (Nanjing, China). Mice were housed in a 12-h dark/light cycle, with a constant temperature of 23 ± 2°C and a relative humidity of 60 ± 2%, and administrated with a Western type diet, which contains 0.25% cholesterol and 15% cacao butter (SDS, Sussex, U.K.) for 6 weeks. Then, the unstable carotid artery plaque model was induced as previously described [23]. In brief, mice were anaesthetized by using isoflurane/O_2_ at a ratio of 2:1, then the common carotid artery and the bifurcation of the carotid artery were carefully separated via 6-0 sutures under the common carotid artery, and ligation was done at about 1 mm away from the bifurcation. The 150 μm needle (Ethicon, 8-0) was placed between the knot and the vessel and the first knot was tightly tied, followed by the second knot being tied in the opposite direction. The common carotid artery was further separated along the proximal end and the same partial ligation was performed again at a distance of 3 mm from the first ligation point. After ligation, the incision was rinsed with sterile saline, and the excess saline was sucked up with cotton balls. Subcutaneous injection of buprenorphine at a dose of 0.1 mg/kg was carried out before and after surgery to alleviate pain [24].

### Mice grouping and drug administration

Following 2 weeks of surgery, the mice were randomly divided into seven groups: (i) 2-week group (2-week after surgery; *n*=6), (ii) water-2-week group (received 2 weeks of water; *n*=10), (iii) MMI-2-week group (received 2 weeks of intragastric administration of MMI (cat. no.: HY-B0208, MedChemExpress, U.S.A.) at a dose of 15 mg/kg, once per day; *n*=10), (iv) LCA-2-week group (received 2 weeks of intragastric administration of LCA (cat. no.: HY-B0399, MedChemExpress) at a dosage of 2 g/kg, once per day; *n*=10), (v) water-5-week group (received 5 weeks of water after 2 weeks of surgery; *n*=10), (vi) MMI-5-week group (received 5 weeks of MMI at a dose of 15 mg/kg, once per day; *n*=10) and (vii) LCA-5-week group (received 5 weeks of LCA at a dosage of 2 g/kg, once per day; *n*=10). LCA was obtained from Lonza Inc. (Allendale, NJ) and MMI was purchased from Sigma-Aldrich (Milwaukee, WI, U.S.A.).

### Plasma and tissue samples collection

At the end of the experimental period, mice were anesthetized via intraperitoneal administration of 1% pentobarbital sodium at a dosage of 65 mg/kg following 12 h overnight fasting. Then, the blood samples from heart were collected and centrifuged for 15 min at a speed of 3000 rpm at 4°C to obtain the plasma samples which were stored at −80°C for further studies. The right common carotid artery was separated under an anatomical microscope and then soaked in 4% paraformaldehyde for 48 h at 4°C. The fresh tissues were then submitted for histopathology analysis or stored with liquid nitrogen quickly. The tissue samples obtained from 5 mice were used for paraffin sections, with others used for frozen sections. Animals were killed using pentobarbital sodium (10 mg/kg).

### Measurement of plasma TMAO levels

Plasma level of TMAO was measured by using the stable isotope dilution LC/MS/MS on an AB Sciex API 5000 triple quadrupole mass spectrometer (Applied Biosystems, U.S.A.) [17]. The plasma samples were mixed with the dedicated liquid mass spectrometry methanol with a ratio of 1:4, and the supernatant was collected after centrifugation at 12,000***g*** for 15 min. Then, 60 μl supernatant was mixed with 1 μl of D_9_-TMAO, the internal standard liquid, followed by detection with a positive ionized sub-mode. TMAO and D_9_-TMAO were monitored by multiple reaction mechanism with parent to daughter transitions, *m/z* 75.9→58.2, *m/z* 85.1→66.0, respectively. Data were analyzed by using Skyline software.

### Analysis of serum lipids

The plasma levels of T-CHO, TG, high-density lipoprotein cholesterol (HDL-C) and LDL-C were measured by using the enzymatic reagent kits (Nanjing Jiancheng Biology Engineering Institute, Jiangsu, China; cat. nos. A111-1, A110-1, A112-1 and A113-1, respectively) according to the descriptions.

### Histopathology analysis

To evaluate the histological characterization of atherosclerotic plaques, the right common carotid arteries were embedded upright in tissue freezing medium and were snap frozen at −80°C. Then, the carotid arteries were cut into 5 μm slices with the help of a Leica CM 1900 cryostat (Leica Biosystems GmbH, Wetzlar, Germany). Hematoxylin and eosin (H&E) staining was conducted to assess the morphological characteristics of the carotid atherosclerotic plaque. Total plaque area was measured by using the ImageJ software. In order to compare the total plaque area fairly, we calculate the plaque area ratio: plaque area ratio = total plaque area/total arterial wall area. Then, Perl's staining (Solarbio, Beijing, China) was performed for ferric iron assessment. For Perl’s staining, carotid samples were incubated for 10 min in a stain containing hydrochloric acid and potassium ferricyanide and then counterstained with eosin. Sirius red and Masson trichrome staining (Sigma-Aldrich) were used to assess Collagen types I and III levels in atherosclerotic lesions, and the sections were analyzed by polarization microscopy. In Sirius red staining, cap thickness was calculated by positive birefringence area underneath polarized light, while lesion height was described as the greatest distance from the arterial wall to the lumen. Cap thickness/lesion height was defined as cap thickness ratio, which could reflect atherosclerotic plaque stability [25].

### Immunohistochemical staining

The paraffin-embedded carotid artery tissues were cut into 4-μm slides, followed by xylene dewaxing and gradient alcohol dehydration. Then, the slides were incubated with 0.3% H_2_O_2_ for 30 min to remove the endogenous peroxides. After that, the sections were blocked with 5% goat serum and incubated with the anti-CD68 antibody (cat. no. 137001, Biolegend, U.S.A.) at 4°C overnight. On the next day, the sections were immersed in horseradish peroxidase (HRP)-conjugated second antibody for 1 h at room temperature. After 2 min of incubation with diaminobenzidine (DAB; cat. no.: ab64261, Abcam, Cambridge, MA, U.S.A.), hematoxylin re-staining was performed. CD68 staining was recorded under a light microscope. The CD68 dyeing area/total plaque area was recorded as CD68 staining.

### Immunofluorescence

Carotid arteries sections with 5 μm in thickness were dewaxed, hydrated and incubated in antigen retrieval solution of 10 mM/l sodium citrate buffer (pH 6), followed by blocking with 3% bovine serum albumin (BSA) in PBS. Then, the sections were incubated overnight at 4°C with the following primary antibodies anti-CD68 (rabbit monoclonal antibody; ab237968, Abcam, Cambridge, MA, U.S.A.), anti-iNOS (mouse monoclonal antibody; No. ab49999, Abcam), anti-Arg1 (mouse monoclonal CoraLite488 antibody; No. CL488-66129, Proteintech, U.S.A.) and anti-cleaved caspase-3 (rabbit polyclonal antibody; No. PA5-105271, Invitrogen, U.S.A.) antibodies with a dilution of 1:50. The sections were then incubated with Donkey Anti-Rabbit IgG H&L (Alexa Fluor® 647) (ab150075, Abcam) or Goat Anti-Mouse IgG H&L (Alexa Fluor® 488) (ab150113) for 1 h at room temperature. Hoechst 33342 (Sigma-Aldrich, U.S.A.) was used to visualize DNA content. Images were taken using a QImaging® EXi Aqua™ monochrome digital camera attached to a Nikon Eclipse 80i Microscope (Nikon).

### Cell culture and treatment

RAW264.7 macrophages and Jurcat T lymphocytes were purchased from American Type Culture Collection (ATCC; Manassas, VA, U.S.A.) and grown in PRIM-1640 medium, with 10% fetal bovine serum (FBS) and 1% penicillin/streptomycin in an incubator with 5% CO_2_ at 37°C. The above regents used in cell culture were all purchased from Thermo Fisher Scientific, lnc (MA, U.S.A.). RAW264.7 cells were incubated with 1, 3, 10, 30 and 100 μM of TMAO for 24 h. In addition, RAW264.7 cells were incubated with 20 ng/ml of IL-4 (Solarbio) for 12 h or 10 ng/ml of IL-13 (Solarbio) for 72 h to induce M2 activation.

### Western blotting analysis

Protein was extracted from cells using lysis buffer (Roche, Shanghai, China) supplemented with 1% protease inhibitor (Solarbio). After centrifugation at 4°C for 25 min, the protein concentrations were determined by using a Bicinchoninic acid Protein Assay kit (Thermo Fisher Scientific) in the light of specification. Then, protein samples were loaded to 10% SDS-polyacrylamide gel and separated by electrophoresis, followed by transformation to polyvinylidene difluoride membranes (PVDF; Millipore, Billerica, MA, U.S.A.). After incubation with 5% non-fat milk for 1 h at room temperature, the membranes were probed with the indicated primary antibodies overnight at 4°C, including anti-actin antibody (No. 3700), purchased from Cell Signaling Technology (MA, U.S.A.) and anti-MerTK antibody (No. ab184086) and anti-SR-BI antibody (No. ab52629), purchased from Abcam. Then, the membranes were incubated with the secondary antibodies for 1 h at room temperature. After washing three times with PBS, the protein signaling was enhanced with ECL reagent (Millipore) and detected on ProfiBlot-48 (Tecan, Switzerland). The gray-scale value analysis was carried out by using ImageJ software.

### Real-time quantitative PCR (RT-PCR)

Total RNA was extracted from cells by using TRIzol reagent based on the manufacturer’s instructions. Then, cDNA was synthesized by using the PrimeScript RT Reagent Kit (Takara, Dalian, China), following by qPCR detection using SYBR Green Master mix (Thermo Fisher Scientific). The relative expression of mRNAs was calculated by 2^−ΔΔCt^ method and normalized to the expression level of β-actin. PCR primers are listed in Table 1.

**Table 1:** Primer sequences

Gene	Forward (5′-3′)	Reverse (5′-3′)
β-Actin	TAGGCGGACTGTTACTGAGC	CTCCTCTTAGGAGTGGGGGT
TNF-α	GGCAGGTTCTGTCCCTTTCA	GGTGGTTTGTGAGTGTGAG
iNOS	GCTCTAGTGAAGCAAAGCCCA	CACATACTGTGGACGGGTCG
IL-6	GCCTTCTTGGGACTGATGCT	TGTGACTCCAGCTTATCTCTTGG
IL-7	TGCTGCACATTTGTGGCTTC	ACCAGGATCAGTGGTTGCTG
Arg1	AGCACTGAGGAAAGCTGGTC	TACGTCTCGCAAGCCAATGT
IL-10	TAAGGCTGGCCACACTTGAG	GTTTTCAGGGATGAAGCGGC
MR	ACAGTTCGACTGGTTGGTGG	ATCCTAGACTCCGGCAGACA
YM1	CAGGTCTGGCAATTCTTCTGAA	GTCTTGCTCATGTGTGTAAGTGA

Abbreviations: Arg1, arginase-1; iNOS, nitric oxide synthase 2; IL-6, interleukin-6; IL-7, interleukin-7; IL-10, interleukin-10; MR, macrophage scavenger receptor 1; TNF-α, tumor necrosis factor-α; YM1, chitinase 3-like 3.

### Efferocytosis assessment

The measurement of the efferocytosis of RAW264.7 cells was performed as described by previously reported [26]. The Jurcat T lymphocytes were made apoptosis by serum withdrawal and UVB (180 mJ/cm^2^) irradiation for 30 min, followed by incubation for 8 h at 37°C. After washing with PBS, the cells were fixed in 4% paraformaldehyde and labeled with CFSE, followed by incubation for 1 h with fresh RAW264.7 cells. Next, the efferocytosis of RAW264.7 cells was assessed by using a fluorescence microscopy. The phagocytic index = (number of phagocytized RAW264.7 cells/number of total cells) × 100%.

### Statistical analysis

Data were presented with means± SD form at least three independent experiments. Statistical analysis was carried out with the help of SPSS23.0 software (IBM Corp.). Comparison was performed using the unpaired Student’s *t*-test and one-way ANOWA. Statistical significance was set as *P*<0.05.

## Results

### Effects of MMI and LCA on the plasma level of TMAO in unstable carotid artery plaque models

**Figure 1:**
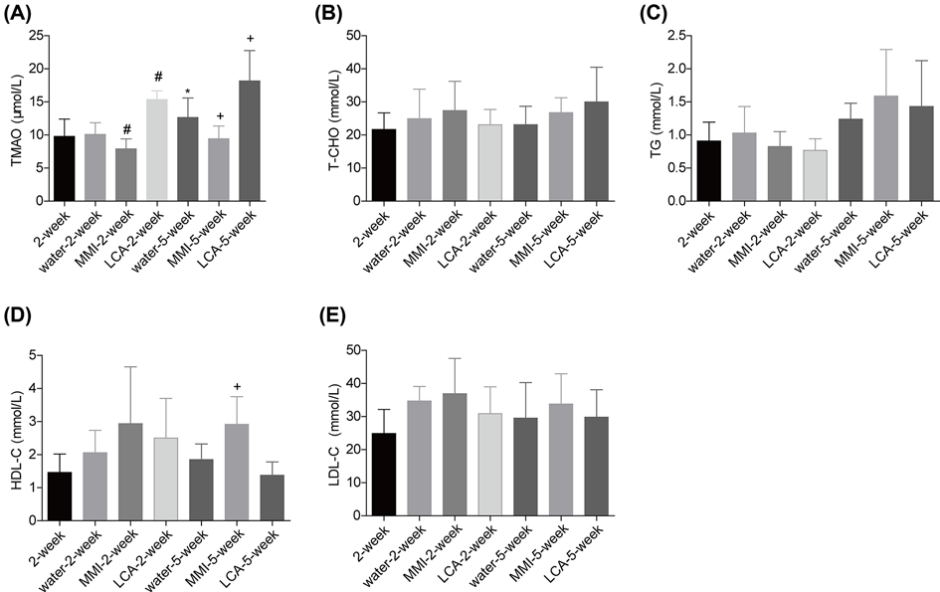
Effect of MMI and LCA on the serum lipid of mice with unstable carotid artery plaques After being anaesthetized, mice common carotid artery and the bifurcation of the carotid artery were separated and ligated to establish the unstable carotid artery plaque models. Then, the mice were divided into 2-week group (*n*=6), water-2-week group (*n*=10), MMI-2-week group (*n*=10), LCA-2-week group (*n*=10), water-5-week group (*n*=10), MMI-5-week group (*n*=10) and LCA-5-week group (*n*=10) and the plasma samples were collected for the following detection. (**A**) Plasma level of TMAO was measured by using the ultrahigh performance liquid chromatography tandem mass spectrometry (UHPLC-MS). (**B–E**) The serum levels of total cholesterol (T-CHO), triacylglycerol (TG), high-density lipoprotein cholesterol (HDL-C) and low-density lipoprotein cholesterol (LDL-C) were measured by using the enzymatic reagent kits (*P<0.05, compared with the 2-week group; #*P*<0.05, compared with the water-2-week group; +*P*<0.05, compared with the water-5-week group).

First, we assessed the effect of MMI and LCA treatment on the levels of serum lipids in the unstable carotid artery plaque models. Compared with the 2-week group, TMAO level was increased gradually after the model establishment as detected in the water-5-week groups (Figure 1A). In comparison with the water administration group, MMI treatment induced a reduction in the plasma levels of TMAO following 2 and 5 weeks of administration, while LCA caused an opposite result after 2 and 5 weeks of administration (Figure 1A). Neither MMI nor LCA treatment showed any obvious influence in the serum liquids of the unstable carotid artery plaque models, including T-CHO (Figure 1B), TG (Figure 1C) and LDL-C (Figure 1E). However, administration of MMI for 5 weeks significantly increased HDL-C level as compared with the water-5-week group (Figure 1D). These results demonstrated that TMAO level was significantly increased in the unstable carotid artery plaque models, which was apparently rescued by MMI administration.

### MMI administration decreased the plaque area in unstable carotid artery plaque models

**Figure 2:**
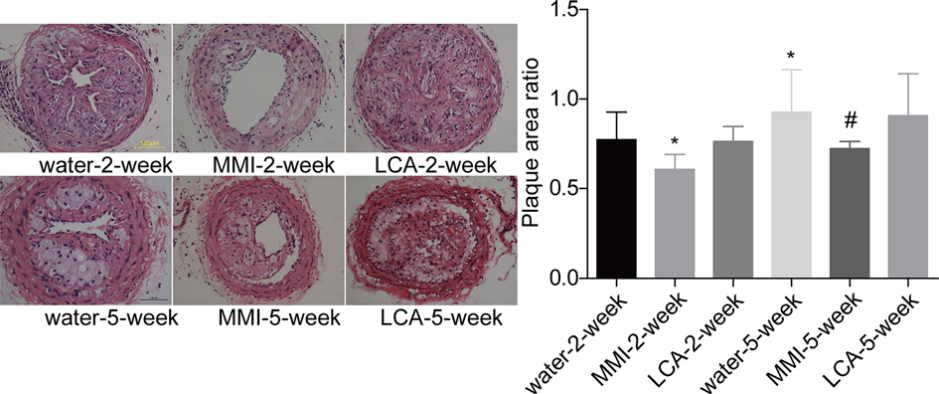
MMI and LCA roles in the plaque area in the unstable carotid artery plaque models Carotid artery plaques were collected from mice in water-2-week group (*n*=10), MMI-2-week group (*n*=10), LCA-2-week group (*n*=10), water-5-week group (*n*=10), MMI-5-week group (*n*=10) and LCA-5-week group (*n*=10), and HE staining was then used to assess the plaque size (*P<0.05, compared with the water-2-week group; #*P*<0.05, compared with water-5-week group).

Then, HE staining was applied to assess the effect of MMI and LCA administration on the plaque area in unstable carotid artery plaque models. Compared with the water-2-week group, the plaque area was significantly increased in the water-5-week group. MMI administration significantly decreased the plaque area as compared with the water administration group for both 2 and 5 weeks of administration, whereas LCA treatment showed no obvious influence in the plaque size (Figure 2). This result demonstrated that MMI administration contributed to the reduction of the plaque area in the unstable carotid artery plaque models.

### MMI administration enhanced the stability of carotid artery plaques

**Figure 3:**
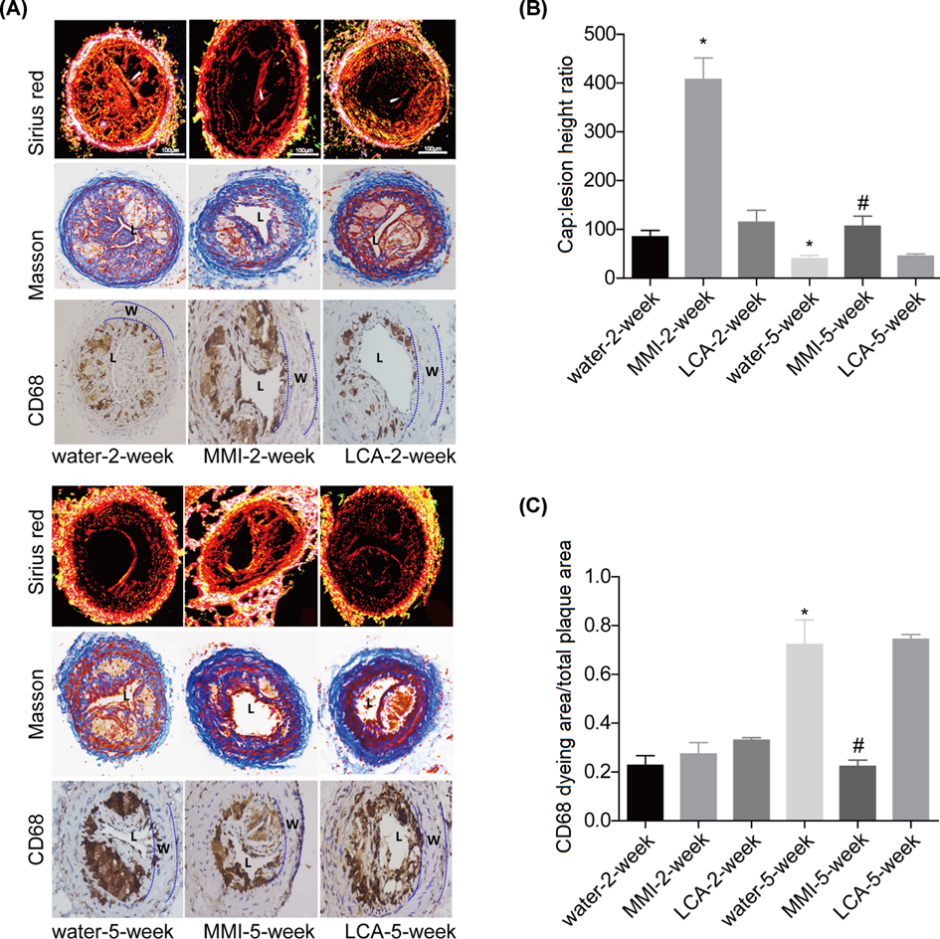
Effects of MMI and LCA on plaque composition in the unstable carotid artery plaque models Carotid artery plaques in mice from the 2-week group (*n*=6), water-2-week group (*n*=10), MMI-2-week group (*n*=10), LCA-2-week group (*n*=10), water-5-week group (*n*=10), MMI-5-week group (*n*=10) and LCA-5-week group (*n*=10) were collected to the following assessments. (**A**) Sirius red staining, Masson trichrome staining and immunohistochemical staining of CD68 were used to assess plaque composition of the right common carotid artery in the unstable carotid artery plaque models (The lumen of the vessel has been indicated as “L”, and the vessel wall has been marked as “W”). (**B**) Bar graph of the Sirius red staining. (**C**) Bar graph of the CD68 staining (*P<0.05, compared with the water-2-week group; #*P*<0.05, compared with water-5-week group).

**Figure 4:**
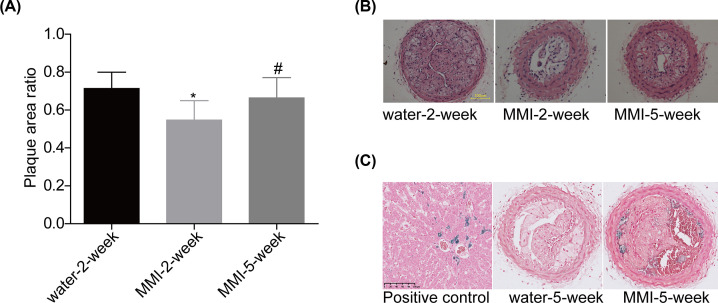
Long-term management of MMI induced intracellular hemorrhage in the unstable carotid artery plaque models (**A** and **B**) HE staining was used to assess the plaque size in different groups. (**C**) Sporadic hemosiderin-laden macrophages were stained by Perl’s staining for iron (stained blue; *P<0.05, compared with the water-2-week group; #*P*<0.05, compared with the MMI-2-week group).

As shown by the Sirius red, Masson trichrome and CD68 staining (Figure 3A), the unstable plaque signs such as large necrotic lipid cores and thin fibrous caps were observed in the water-2-week and water-5-week groups, whereas MMI administration for 2 and 5 weeks obviously increased the thickness of the fibrous cap and reduced the size of the necrotic lipid cores, especially in the MMI-2-week group. Compared with the water administration group, LCA administration further decreased the thickness of the fibrous cap and increased the size of the necrotic lipid cores. In comparison with water administration group, MMI administration significantly increased the stability of carotid artery plaques which was assessed by the ration of fiber cap area to lipid core length and decreased the CD68 content, whereas LCA administration further decreased the stability of carotid artery plaques and increased CD68 content with no significant difference (Figure 3B,C). MMI administration for 2 weeks showed better role in increasing the stability of carotid artery plaques than 5-week group. Further analysis showed that long time administration of MMI induced intraplate hemorrhage of carotid artery plaques in 5 mice form a total of 10 mice, as shown by the HE staining and (Figure 4A,B) and Perl’s staining (Figure 4C). These results demonstrated that MMI administration enhanced the stability of carotid artery plaques but induced hemorrhage for long time administration.

### TMAO impairs M2 polarization

**Figure 5:**
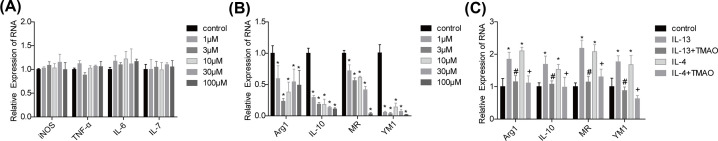
Effects of TMAO on the M1 and M2 polarization of RAW264.7 cells (**A** and **B**) The expression levels of M1 (iNOS, TNF-α, IL-6 and IL-7) and M2 (Arg1, IL-10, MR and YM1) macrophagocyte markers in RAW264.7 cells treated with TMAO (0, 1, 3, 10, 30, 100 μM of 24 h) were determined by using RT-PCR. (**C**) The mRNA levels of Arg1, IL-10, MR and YM1 were detected by RT-PCR in RAW264.7 cells treated with IL-4 (20 ng/ml; 12 h) or IL-13 (10 ng/ml; 72 h) and TMAO (100 μM; 24 h; *n*=3, *P<0.05, compared with the control group; **#***P*<0.05, compared with the IL-13 group; **+***P*<0.05, compared with the IL-4 group).

**Figure 6:**
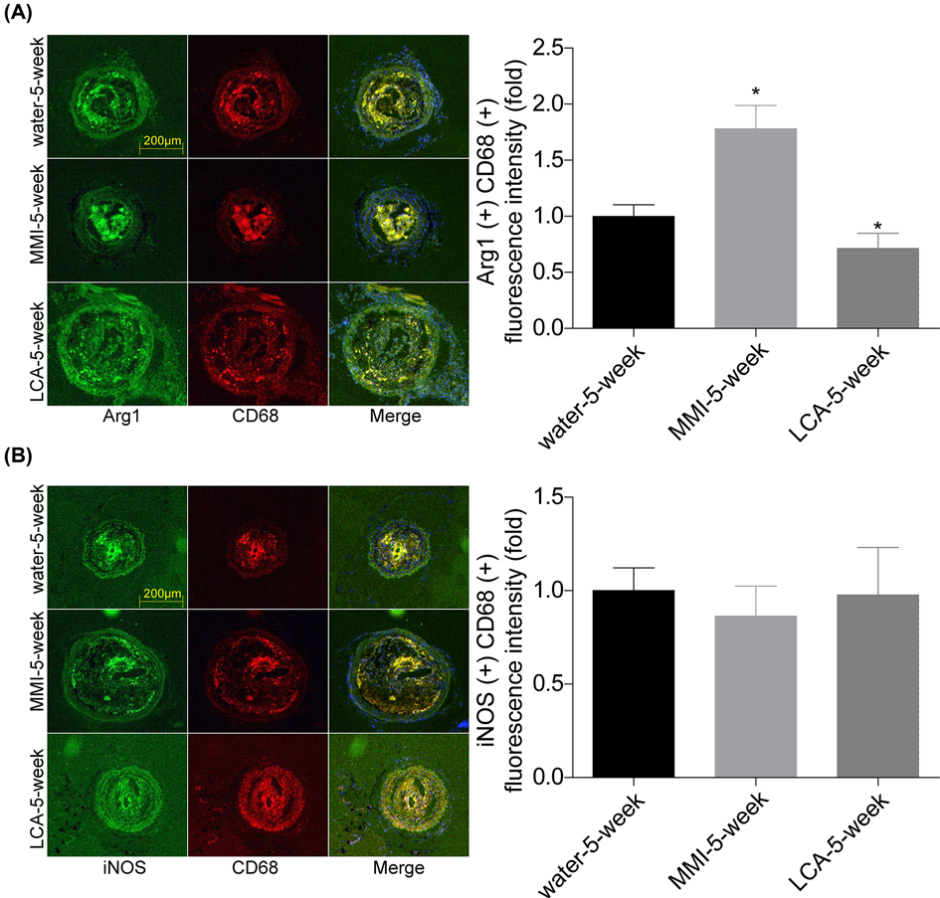
MMI administration promoted M2 polarization and inhibited efferocytosis *in vivo* (**A** and **B**) Immunofluorescence staining was used to evaluate the expression and location of Arg1, CD68 and iNOS in carotid arteries samples of mice in water-5-week, MMI-5-week and LCA-5-week groups. The co-locations of CD68 and Arg1/iNOS were quantitated (*P<0.05, compared with the water-5-week group).

Additionally, we explored the underlying mechanism of TMAO in the stability of carotid artery plaques. Compared with the control group, the mRNA levels of M1 markers, including iNOS, TNF-α, IL-6 and IL-7 showed no obvious influence in cells treated with different concentrations of TMAO (Figure 5A). However, TMAO treatment significantly increased the expression levels of M2 markers, including Arg1, IL-10, MR and YM1 (Figure 5B). To further explore TMAO role in M2 polarization, RAW264.7 cells were treated with IL-4 or IL-13 to induce M2 polarization. As shown in Figure 5C, TMAO treatment significantly decreased the mRNA levels of Arg1, IL-10, MR and YM1 induced by IL-4 and IL-13 treatment. A similar phenomenon was observed in mice carotid arteries. Compared with the water-5-week group, the co-expression level of CD68 and Arg1 was increased in MMI-5-week group and decreased in LCA-5-week group (Figure 6A), while iNOS level showed no obvious change between the three groups (Figure 6B). These results demonstrated that TMAO inhibited M2 polarization.

### TMAO impedes efferocytosis

**Figure 7:**
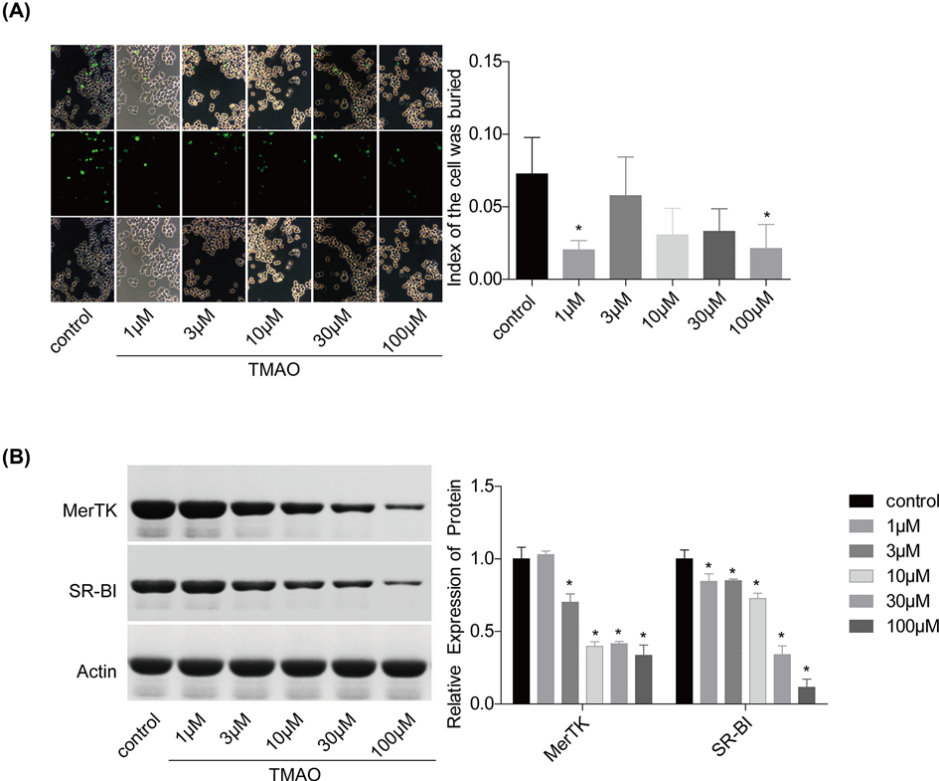
Effects of TMAO on the efferocytosis of RAW264.7 cells (**A**) The efferocytosis of RAW264.7 cells with different treatments (0, 1, 3, 10, 30, 100 μM of TMAO; 24 h) were assessed. (**B**) The expression levels of MerTK and SR-BI were measured by Western blotting (*n*=3, *P<0.05, compared with the control group).

**Figure 8:**
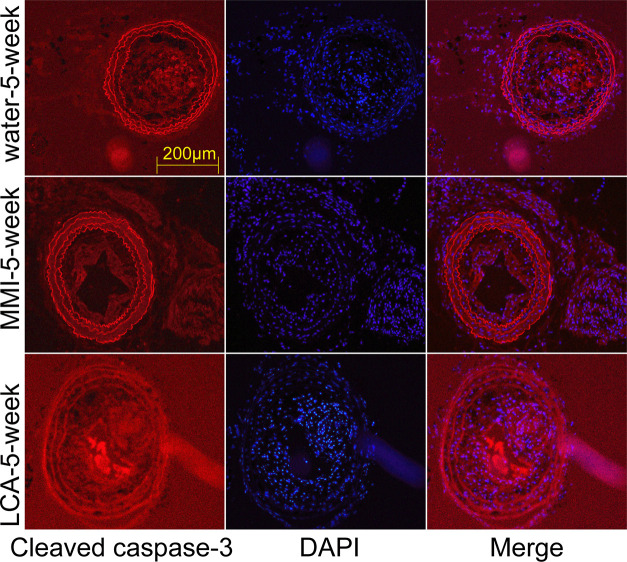
MMI administration inhibited efferocytosis *in vivo* Immunofluorescence staining was used to evaluate the expression of cleaved caspase-3 in carotid arteries samples of mice in water-5-week, MMI-5-week and LCA-5-week groups.

Furthermore, we also explored TMAO effect on the efferocytosis of RAW264.7 macrophagocytes. Compared with the control group, TMAO treatment significantly decreased the efferocytosis of RAW264.7 macrophagocytes (Figure 7A), with decreased expression levels of MerTK and SR-BI (Figure 7B). Moreover, the expression level of cleaved caspase-3 (an efferocytosis marker) was increased in LCA group and decreased in MMI group as compared with the water-5-week group (Figure 8). These results showed that TMAO treatment inhibited efferocytosis of RAW264.7 cells *in vitro* and *in vivo*.

## Discussion

Although atherosclerosis is a relatively benign process of slow, it may be abruptly complicated by rupture or erosion of an atherosclerotic plaque with an overlying thrombosis, leading to acute ischemic event [27]. Noticeably, atherosclerosis is one of the most common causes of morbidity and mortality worldwide [28]. Increasing evidence has demonstrated that TMAO makes a significant contribution to the development of atherosclerotic plaques [17,29]. Herein, we found that reduction of TMAO through MMI administration significantly reduced the carotid plaque size and enhanced the stability of carotid plaque.

Studies have demonstrated that plasma TMAO level is elevated in atherosclerosis [15,30], and increased TMAO concentrations predict incident risks in cardiovascular diseases [29,31]. LCA as an abundant nutrient in red meat was reported to induce TMAO production in both mice and human [17,32] and accelerate atherosclerosis in mice via gut microbiota [17]. Clinical studies have found a striking correlation between TMAO and cardiovascular disease with inhibition of TMAO production as a potential method for atherosclerosis treatment [33–35]. However, some researchers hold the opposite view. For instance, a TMAO-rich fish diet has been shown to reduce the risk of cardiovascular disease [36]. DiNicolantonio et al. [37] also suggests that a diet rich in LCA may serve as a secondary preventative measure for cardiovascular disease. Collins et al. [38] demonstrated that administration of LCA induced significant increase in plasma TMAO levels in ApoE^−/−^ mice expressing hCETP (human cholesteryl ester transfer protein), resulting in a significant reduction in aortic lesion size in both aortic root and thoracic aorta, indicating that TMAO inhibits aortic lesion formation and may have a protective role against atherosclerosis development in humans. However, Collins et al. [38] also showed that administration of MMI, an inhibitor of FMO which is the major hepatic enzyme involved in the conversion of TMA to TMAO metabolite [40] also induced reduction in aortic lesion size, which may be caused by the antioxidant and anti-inflammatory effects reported for MMI [39,40]. In the present study, we explored the effects of LCA and MMI in the lesion size of carotid atherosclerotic plaques in ApoE^−/−^ mice. The results showed that MMI administration at a dosage of 15 mg/kg for 2 and 5 weeks significantly decreased TMAO level, and then induced a significant reduction in lesion size. Our results also suggest a beneficial role of MMI in the inhibition of carotid artery plaques formation in ApoE^−/−^ mice. Administration of LCA at a dosage of 2 g/kg significantly increased the plasma level of TMAO, but LCA administration showed no significant effect on the lesion size. ApoE^−/−^ mice expressing hCETP increases the cholesterol outflow, which may cause the protective role of LCA against atherosclerosis development [38].

Additionally, Tan et al. [41] recently reported that TMAO was a new marker of atherosclerotic plaque rupture in ST-segment elevation myocardial infarction patients, suggesting that TMAO may be associated with plaque stability. Herein, we observed that MMI administration significantly enhanced stability of carotid atherosclerotic plaques with increased collagen content and reduced macrophage content, indicating a protective role of MMI in keeping the stability of carotid atherosclerotic plaques. However, long time administration of MMI for 5-week induced intraplaque hemorrhage which was not found in MMI-2-week group, indicating a side effect of long-time administration of MMI. It has been reported that MMI is metabolized by cytochrome P450 (CYP450) and FMO enzyme to metabolites which are suspected to be cytotoxic. I think this cytotoxicity may be the major cause for the side effect of MMI long time administration in carotid atherosclerotic plaques.

In mechanism, we explored the effect of TMAO on the macrophage polarization and efferocytosis in RAW264.7 cells. Our results demonstrated that TMAO treatment significantly inhibited the M2 polarization and efferocytosis of RAW264.7 cells *in vitro*, with no obvious effect on the M1 polarization. These results suggested that TMAO triggered the instability of carotid atherosclerotic plaque might through impeding macrophage M2 polarization and efferocytosis.

## Conclusion

The present study demonstrates that MMI-induced TMAO reduction enhances the stability of carotid atherosclerotic plaques, which might be induced by the promotion of macrophage M2 polarization and efferocytosis. Collectively, this study demonstrates that MMI might be used as an effective drug to enhance the stability of carotid atherosclerotic plaques.

## Data Availability

All supporting data are included within the main article.

## Abbreviations

Arg1: arginase-1
H&E: hematoxylin and eosin
hCETP: human cholesteryl ester transfer protein
IL: interleukin
iNOS: nitric oxide synthase 2
LCA: L-carnitine
MMI: methimazole
MR: macrophage scavenger receptor 1
TMAO: trimethylamine N-oxide
TNF-α: tumor necrosis factor-α
